# Supplementary material for: SARS-CoV-2 infection: Initial viral load (iVL) predicts severity of illness/outcome, and declining trend of iVL in hospitalized patients corresponds with slowing of the pandemic
Source: PLoS One. 2021 Sep 16;16(9):e0255981. doi: 10.1371/journal.pone.0255981 (PMC8445469; doi:10.1371/journal.pone.0255981)
Supplement: S1 Table — (DOCX) [file pone.0255981.s001.docx]

| Variable | Intubation | | | Mortality | | |
| --- | --- | --- | --- | --- | --- | --- |
|  | Yes | No | P value | Yes | No | P value |
| Age |  |  |  |  |  |  |
| 18-39 | 6 (5.1) | 16 (4.7) | 0.917 | 2 (1.5) | 20 (6.1) | **< 0.001** |
| 40-65 | 44 (37.3) | 122(35.6) |  | 31 (23.5) | 135 (41.0) |  |
| > 65 | 68 (57.6) | 205(59.8) |  | 99 (75.0) | 174 (52.9) |  |
| Gender |  |  |  |  |  |  |
| Male | 61 (51.7) | 182 (43.1) | 0.798 | 76 (57.6) | 167 (50.8) | 0.185 |
| Female | 57 (48.3) | 161 (46.9) |  | 56 (42.4) | 162 (49.2) |  |
| Race |  |  |  |  |  |  |
| African American | 99 (83.9) | 263 (76.7) | 0.243 | 110 (83.3) | 252 (76.6) | 0.219 |
| White | 8 (6.8) | 30 (8.7) |  | 10 (7.6) | 28 (8.5) |  |
| Other | 11 (9.3) | 50 (14.6) |  | 12 (9.1) | 49 (14.9) |  |
| Comorbidities |  |  |  |  |  |  |
| BMI |  |  |  |  |  |  |
| < 18 | 2 (1.7) | 10 (2.9) | 0.135 | 3 (2.3) | 9 (2.7) | 0.994 |
| 18-25 | 23 (19.5) | 99 (28.9) |  | 35 (26.5) | 87 (26.4) |  |
| 25-30 | 35 (29.7) | 100 (29.2) |  | 39 (29.5) | 96 (29.2) |  |
| >30 | 58 (49.2) | 134 (39.1) |  | 55 (41.7) | 137 (41.6) |  |
| Hypertension | 91 (77.1) | 264 (77.0) | 0.973 | 103 (78.0) | 252 (76.6) | 0.741 |
| Diabetes | 60 (50.8) | 143 (41.7) | 0.15 | 54 (40.9) | 149 (45.4) | 0.392 |
| Chronic Kidney Disease | 40 (33.9) | 96 (28.0) | 0.225 | 33 (25.0) | 103 (31.3) | **0.085** |
| Coronary artery disease | 24 (20.3) | 75 (21.9) | 0.728 | 29 (22.0) | 80 (21.3) | 0.870 |
| Heart failure | 13 (11.0) | 32 (9.3) | 0.594 | 13 (9.8) | 32 (9.7) | 0.968 |
| COPD | 19 (16.1) | 64 (18.7) | 0.533 | 26 (19.7) | 57 (17.3) | 0.549 |
| Smoking | 18 (15.3) | 73 (21.3) | 0.156 | 25 (18.9) | 66 (20.1) | 0.785 |
| Viral load |  |  |  |  |  |  |
| Low viral load | 44 (37.3) | 151 (44.0) | 0.065 | 31 (23.5) | 164 (49.8) | **< 0.001** |
| Intermediate viral load | 40 (33.9) | 128 (37.3) |  | 50 (37.9) | 118 (35.9) |  |
| High viral load | 34 (28.8) | 64 (18.7) |  | 51 (38.6) | 47 (14.3) |  |

**S1 Table:** Univariate analysis of factors associated with increased risk for intubation and mortality
