# Supplementary figures and images for: SARS-CoV-2 infection: Initial viral load (iVL) predicts severity of illness/outcome, and declining trend of iVL in hospitalized patients corresponds with slowing of the pandemic
Source: PLoS One. 2021 Sep 16;16(9):e0255981. doi: 10.1371/journal.pone.0255981 (PMC8445469; doi:10.1371/journal.pone.0255981)

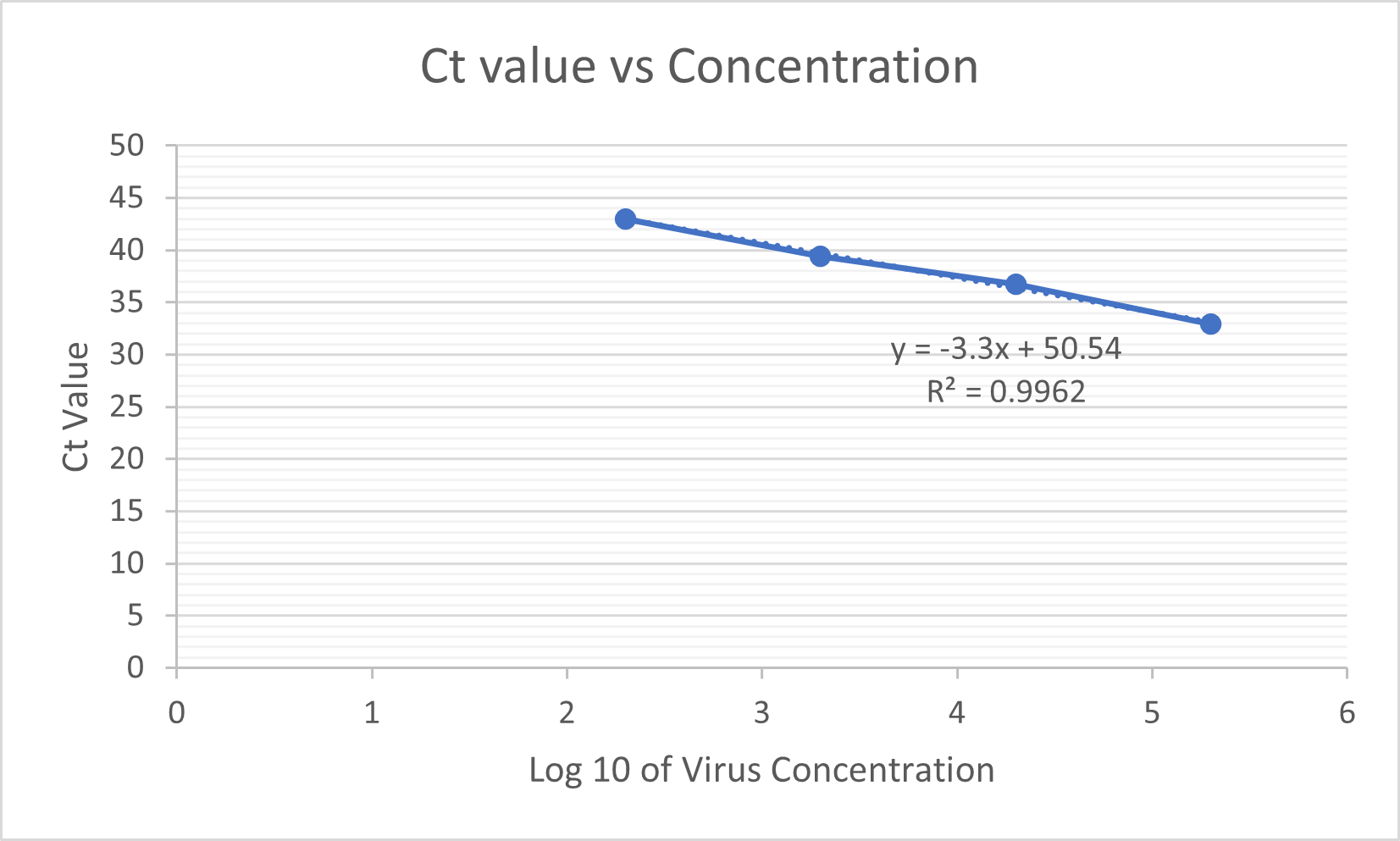

Supplement: S1 Fig — (TIF) [file pone.0255981.s002.tif]

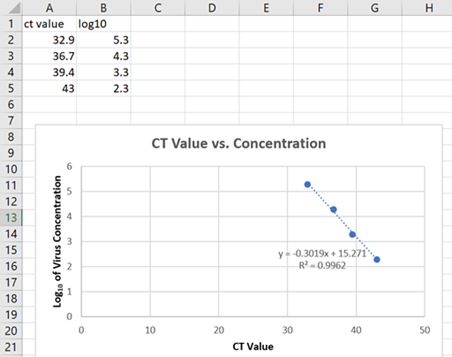

Supplement: S2 Fig — (PNG) [file pone.0255981.s003.png]
