# Supplementary material for: SARS-CoV-2 infection: Initial viral load (iVL) predicts severity of illness/outcome, and declining trend of iVL in hospitalized patients corresponds with slowing of the pandemic
Source: PLoS One. 2021 Sep 16;16(9):e0255981. doi: 10.1371/journal.pone.0255981 (PMC8445469; doi:10.1371/journal.pone.0255981)
Supplement: Attachment — Submitted filename: Response to reviewers.docx [file pone.0255981.s005.docx]

Dear editor
Tai-Heng Chen, M.D.
PLOS One

Thank you for considering our manuscript for publication.
We would also like to thank the reviewers for their valuable and constructive comments.

Kindly find below in bold our point-by-point response to the academic editor and reviewers’ comments:

**Reviewer #2:**

1. Thank you for addressing all comments. However, I still can not see the evidence of any standard curve to partially validate the quantification of VL by the QPCR. IT is mentioned that 1 to 10 dilution was used to draw such a standard curve and R^2 was 0.9, but no data has been provided. The source or specifications of the commercial virus sample is not clear as well.

**Thank you for your comment. Please find below our answer to comments #1 and #2 respectively:**

**Source and specifications of the commercial virus sample:**

**In order to determine the performance of Cepheid Genexpert SARS-CoV-2 assay we used quantified standard materials that were obtained from Exact Diagnostics (**[**www.exactdiagnostics.com**](http://www.exactdiagnostics.com)**). The product from Exact diagnostics is EDX SARS-CoV-2 Standard that is manufactured with synthetic RNA transcripts containing five gene targets (E, N, ORF1ab, RdRP and S Genes of SARS-CoV-2). The product allows laboratories to validate/verify the entire process of the assay including extraction, amplification, and detection. The EDX SARS-CoV-2 Standard contains E, N, ORF1ab, RdRP and S genes that are each quantitated at 200,000 cp/mL using Bio-Rad Digital Droplet PCR (ddPCR).**

**The other commercially available source that we used was Zeptometrix NATtrol™ (SARS-CoV-2) External positive Control. It was used as well for the validation/verification studies and in determination of the lower limit of detection of our SARS-CoV-2 assay.**

**This control is a quantified source for SARS-CoV-2 and has a concentration of 50,000 copies/ml.**

**We also received SARS-CoV-2 virus from BEI resources repository (**[**www.niaid.nih.gov/research/bei-resources-repository**](http://www.niaid.nih.gov/research/bei-resources-repository) **after signing a material transfer agreement. Being able to obtain the SARS-CoV-2 was a big factor in quick validation/verification of our SARS-CoV-2 assay at the very early stage of COVID-19 pandemic which allowed our laboratory to start testing patient samples for COVID-19 in March of 2020.**

**Standard curve:
We are attaching a picture of the standard curve below. We are also attaching this figure in the submission system for reference.**

**After repeating the experiment, we have made few changes pertaining to the correlation between the viral loads and corresponding Ct values. The changes made are highlighted below, and these do not affect our results or conclusions. (Lines 110-114)**

**At 2x10^2^, 2x10^3^ and 2x10^4^ viral particles, the Ct values were 43, 39.4 and 36.7 respectively. We designated high, intermediate, and low VL samples to have a Ct value of ≤ 25, 26-36, and ≥ 37 consecutively, therefore, assuming a linear relationship between the Ct value and target concentration, samples with a Ct value of 26 should have a VL of 2x10^7^ while samples with a Ct value of 36 should have a VL of 2x 10^4^ approximately.**

**The viral load categories (low, intermediate and high) were chosen based on the Ct values of <25, 26-36 and > 37 respectively. Other comparable categories of viral load are used in the literature. (Rabaan AA, et al. *Diagnostics (Basel)*. 2021;11(6):1091 and Rao SN, et al. Infect Dis Ther 2020;9(3):573-586. doi:10.1007/s40121-020-00324-3.**

**Correlating a Ct value to a viral load in our manuscript is used only to provide perspective to the readers. Moreover, the association between Ct value and mortality was also demonstrated on multivariate logistic regression when Ct values were analyzed as continuous variables.**

2. I am not sure, but I think the added statistical analysis is not accurate. I appreciate testing for normality of data and comparing age and Ct in outcome groups by Mann-Withney U test, but I do not understand how and on what variables the linear regression model was fitted. the equation for table 3, Death = 0.431 + 0.006*Age - 0.016*Ct value, looks like a logistic regression equation to me, whereas by "Death" the authors mean the probability of death, but it is not mentioned in the method section. Beside, in the same table, while age and Ct value are parametric variables, CKD must be nominal and it is not clear to me by which method this p value has been calculated.

**Based on your recommendations, we performed a multivariate logistic regression model using age and Ct value as continuous variables and added a paragraph to the manuscript. (Lines 234-240).**

**Using multiple logistic regression, both age and Ct value were found to be significantly associated with mortality, such as every 1-year increase in age results in 3.5% increase in odds of death and every 1 unit increase in Ct value decreases the odds of death by 8%. The negative association between Ct and mortality reflects the fact that lower Ct values indicate higher viral load (Table 3).**

**Table 3: Multiple logistic regression of independent predictors of in-hospital mortality using Age and Viral Load as continuous variables.**

|  | **Mortality** | |
| --- | --- | --- |
| **Variables** | **Odds Ratio (95% CI)** | **p-value** |
| **Age** | **1.035 (1.018-1.053)** | **<0.001** |
| **Ct Value** | **0.921 (0.894-0.949)** | **<0.001** |

**Reviewer #3:**

My comments have been addressed by the authors, but regarding the reviewer #2 comments 1 &2 it is suggested that brief explanations similar to the response to the reviewer about the Cepheid GenExpert instrument system be given in the text.

**Thank you for your feedback. We added a paragraph to the methods section briefly explaining how our assay does not contain an internal control but instead, the SPC utilized by the GeneXpert Xpress System instrument serves to confirm adequate processing of the sample and to ensure that the RT-PCR reaction conditions are appropriate (Lines 102- 105).**

Sincerely,

Pranatharthi Chandrasekar, MD

Division of Infectious Diseases

Wayne State University School of Medicine, Detroit, Michigan

Phone: 313-745-7105 / Fax: 313-993-0302
